# Unveiling a Rare and Aggressive Bladder Tumor: A Case Report of Plasmacytoid Urothelial Carcinoma

**DOI:** 10.7759/cureus.105275

**Published:** 2026-03-15

**Authors:** Fatima Zahra Bouayed, Mohammed Amine Guerrouaz, Hanane Sebbar, Ahmed BenSghier, Soufiane Berhili, Mohamed Moukhlissi, Loubna Mezouar

**Affiliations:** 1 Radiation Oncology, Faculty of Medicine and Pharmacy, Mohammed First University, Oujda, MAR; 2 Radiation Therapy, Mohammed VI University Hospital, Oujda, MAR; 3 Pathology, Mohammed VI University Hospital, Oujda, MAR; 4 Radiation Oncology, Mohammed VI University Hospital, Oujda, MAR; 5 Radiotherapy, Mohammed VI University Hospital, Oujda, MAR; 6 Radiation Oncology, Mohammed VI University Hospital, oujda, MAR

**Keywords:** concomitant chemo-radiotherapy, plasmacytoid, plasmacytoid urothelial carcinoma, plasmacytoid variant urothelial carcinoma, radiation oncoolgy

## Abstract

In 1991, Sahin et al. reported the first documented case of plasmacytoid urothelial carcinoma (PUC). A rare and highly aggressive subtype that was recently incorporated into the WHO classification of urothelial carcinomas, representing 1-3% of cases of bladder cancers. The tumor cells are characterized by eccentrically placed nuclei with abundant eosinophilic cytoplasm and demonstrate positive immunostaining for CD138, and this variant is generally linked to an unfavorable prognosis. In this article, we present the case of a 61-year-old male diagnosed with PUC managed with concurrent chemoradiotherapy.

## Introduction

Urothelial carcinoma accounts for nearly 90% of bladder cancer, with a higher incidence in men than in women [1]. Plasmacytoid urothelial carcinoma (PUC ) is an uncommon and highly malignant subtype that was recently included in the WHO classification of urothelial carcinomas [2]. The first ever documented case dates back to 1991 by Sahin et al. [3]. Because it is often diagnosed at an advanced stage, this variant is typically linked to an unfavorable prognosis and an aggressive clinical behavior [4]. We present the case of a 61-year-old male with plasmacytoid urothelial carcinoma managed with concurrent chemoradiotherapy.

## Case presentation

**Figure 1:**
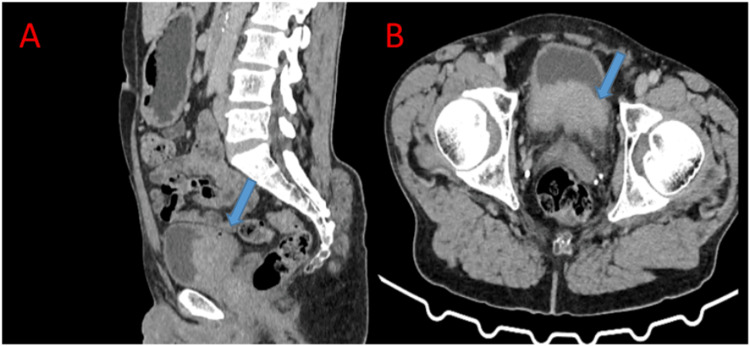
CT urography demonstrates infiltrative bladder wall thickening involving the ureteral orifices and the bladder base, with extension into the perivesical fat A: sagittal section; B: axial section.

We report the case of a 61-year-old man with a history of chronic kidney disease, benign prostatic hyperplasia, and a history of smoking and alcohol consumption, who presented to our radiation oncology department with moderate pollakiuria and hematuria. A uro-computed tomography scan revealed bilateral ureteropyelocaliceal dilatation, more pronounced on the right side, secondary to an infiltrative bladder lesion involving the ureteral orifices, with associated perivesical fat infiltration and small right external iliac lymph nodes (Figure 1).

A follow-up CT scan of the chest, abdomen, pelvis, and neck was performed and did not reveal any signs of metastasis. The patient subsequently underwent cystoscopic evaluation followed by transurethral resection of the bladder tumor, and a right nephrostomy was placed to relieve urinary obstruction.

Histopathological examination revealed an infiltrating urothelial carcinoma with plasmacytoid features. Microscopically, the tumor was composed of discohesive malignant cells arranged in solid sheets and cords, exhibiting marked nuclear atypia, hyperchromatic nuclei, prominent nucleoli, abundant eosinophilic cytoplasm, and frequent mitotic figures. Tumor cells infiltrated both the lamina propria and the muscularis propria of the bladder wall. Vascular emboli were present, with no evidence of perineural invasion.

**Figure 2:**
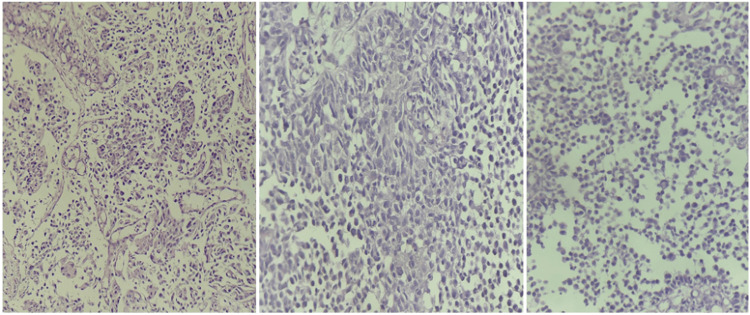
Histopathological examination Histological images showing plasmacytoid urothelial carcinoma of the bladder, composed of medium-sized tumor cells with eosinophilic cytoplasm and eccentrically located nuclei, giving them a plasmacytoid appearance. The tumor cells are dispersed singly within a loose stroma. (Hematoxylin and eosin staining, magnification ×40).

Immunohistochemical analysis demonstrated positive staining of the plasmacytoid tumor cells for CD138. These findings are consistent with a diagnosis of high-grade infiltrating plasmacytoid urothelial carcinoma (Figure 2 ). 

During the pre-chemotherapy workup, a previously undiagnosed case of heart failure with reduced left ventricular ejection fraction (30%) was discovered, and the patient was started on optimized medical therapy. The case was discussed in a multidisciplinary tumor board. Chemotherapy and surgery were both considered, but because of the patient’s severe cardiac dysfunction (ejection fraction 30%) and chronic kidney disease, these options were deemed unsuitable. Concomitant chemoradiotherapy was therefore recommended.

The patient underwent external three-dimensional conformal radiotherapy targeting the tumor, receiving a total dose of 55 Gy delivered in 20 fractions of 2.75 Gy each, administered concomitantly with chemotherapy based on gemcitabine at 100 mg/m², of which the patient received two cycles, each preceded by a thorough clinical and radiological evaluation, with rigorous monitoring of cardiac and renal function. Over the six months after completing radiation therapy, the patient remained well and showed no new clinical issues

## Discussion

Approximately 1 to 3% of cases of bladder cancer are plasmacytoid urothelial carcinoma [5]. This infrequently encountered variant is commonly linked to an aggressive clinicopathologic pattern. A particular tumor morphology and an unusual clinical presentation further distinguish it [5,6]. The majority of patients exhibit hematuria and other irritative urinary symptoms, frequently seen in elderly male patients with a smoking history [6]. In 1991, Sahin et al. documented the very first case of PUC in a 63-year-old man whose tumor displayed a myeloma-like histologic appearance and was associated with lytic bone lesions [7]. And this rare subtype was not adopted by the World Health Organization (WHO) classification of urothelial carcinomas until recently [2]. Microscopically, PUC display eccentrically placed nuclei with abundant eosinophilic cytoplasm and stain positively for CD138 [8,9]. Tumor cells frequently infiltrate the lamina propria and muscularis mucosae, demonstrating a strong tendency for aggressive local extension and metastatic dissemination [10], disclosing its aggressive clinical course as well as treatment challenges. Kohno et al. documented the first instance of an entire pathological response in plasmacytoid urothelial carcinoma in a patient with cT4N0M0 disease who benefited from radical cystectomy after neoadjuvant chemotherapy [11]. Yet, there is still a dearth of data in the literature. Hence, conclusions about the ideal management methods need to be considered with caution, particularly in clinical scenarios where some treatment options may be contraindicated; nonetheless, the course of treatment is comparable to that for other urothelial carcinomas, involving radiotherapy, chemotherapy, and surgery [1]. One of the main characteristics of plasmacytoid urothelial carcinoma is its strong tendency to spread to the peritoneum [8]. Some studies have suggested that elevated serum CA-125 levels may precede peritoneal recurrence, though there is currently insufficient evidence to support this theory [8]. And according to a recent study, plasmacytoid urothelial carcinoma and conventional urothelial carcinoma possess a similar mutational profile, with the primary distinction being the presence of CDH1 gene mutations, which are associated with loss of E-cadherin expression [12]. Moreover, telomerase reverse transcriptase (TERT) promoter mutations have been found in about 40% of plasmacytoid cases, which is consistent with earlier research like that presented by Palsgrove, who found that tumors with plasmacytoid characteristics frequently displayed these mutations [12]. In this case, systemic chemotherapy and radical surgery were first deemed to be the standard course of treatment. However, these approaches were inappropriate because of the patient's severe cardiac dysfunction (ejection fraction of 30%) and chronic kidney disease, which significantly elevated the risk of perioperative and treatment-related complications. In a broader sense, substantial comorbidities can limit therapeutic options and make patient management more challenging.

## Conclusions

PUC remains a rare entity that continues to pose significant challenges during the course of treatment. A multidisciplinary approach is crucial to improve therapeutic outcomes and to achieve better prognostic results, and further investigations are needed to define better management strategies , particularly for patients for whom standard treatment options are inadequate .
